# Sensitisation patterns and allergy outcomes in pregnant women living in the urban area

**DOI:** 10.1186/s13223-021-00547-0

**Published:** 2021-05-10

**Authors:** Hanna Danielewicz, Anna Dębińska, Grzegorz Myszczyszyn, Anna Myszkal, Lidia Hirnle, Anna Drabik-Chamerska, Danuta Kalita, Andrzej Boznański

**Affiliations:** 1 1st Department of Pediatrics, Allergology and Cardiology, Wroclaw Medical University, ul. Chalubinskiego 2a, 50-368 Wroclaw, Poland; 2 1st Department of Gynecology and Obstetrics, Wroclaw Medical University, ul. Chalubinskiego 5, 50-368 Wroclaw, Poland; 3 1st Department of Gynecology and Obstetrics, University Hospital of Jan Mikulicz-Radecki in Wroclaw, ul. Chalubinskiego 5, 50-368 Wroclaw, Poland

**Keywords:** Sensitisation, Atopy, Pregnancy, Allergy

## Abstract

**Background:**

Worldwide, allergy affects more than one billion people, with particularly rising prevalence in industrialised areas. Specifically, young adults appear to be predominantly targeted for an allergy diagnosis. Allergic diseases in pregnancy are mainly pre-existing but could also occur de novo. The immunological changes while pregnant, with increased Th2 lymphocyte activity, can facilitate allergen sensitisation.

**Objective:**

The aim of this study was to evaluate the pattern of specific IgE (sIgE) sensitisation to common inhalant and food allergens in pregnancy, and assess its relationship to self-reported allergic disease.

**Methods:**

We assessed 200 pregnant women, aged 20–38 years (mean age = 29 years), participant of ELMA (Epigenetic Hallmark of Maternal Atopy and Diet) study, living in a metropolitan area, with no pregnancy associated metabolic complications, for total IgE and allergen specific IgE to 20 allergens.

**Results:**

48% of pregnant women were sensitised to at least one allergen, at a cut-off point of 0.35 kU/L and they were assigned as atopic. However 42% in atopic group were not reporting any allergic disease. The most common inhalant allergens were: pollen (24.5%) and animal dander (23.5%). The most common food allergens were: cow’s milk (5.5%) and apples (4.5%). 7.5% of women reported asthma, 21.5% allergic rhinitis, 11.5% atopic dermatitis and 18.5% food allergy. 8.5% of were taking medication for asthma or allergies. Atopic dermatitis had the highest tendency to become more severe during pregnancy. Total IgE values were significantly higher in atopic women.

**Conclusions:**

Allergic sensitisation is a common phenomenon in pregnancy. Some sensitisations could be asymptomatic. Further studies should investigate if sensitisation in mothers confers risks for immune alterations in their children.

**Supplementary Information:**

The online version contains supplementary material available at 10.1186/s13223-021-00547-0.

## Introduction

Worldwide, allergy affects more than one billion people [1]. Specifically, young adults appear to be predominantly targeted for an allergy diagnosis. In pregnancy, allergic disease influences both the mother and child, however little is known if allergies confer adverse pregnancy outcomes, such as stillbirth or preterm delivery. Different studies have suggested some risk towards unfavourable pregnancy outcomes for asthma patients, but unexpectedly also protection for allergic rhinitis and atopic dermatitis patients [2]. This relation could be also mirroring some other moderating factors like level of education. Higher education and socio-economic status are associated with higher risks of allergy but also with lower risk for poor pregnancy outcome [3, 4]. Pregnancy is a physiologically Th2 dominant state. The source of Th2 cytokines are gestational tissues, requiring progesterone and oestrogen to amplify the effects [5, 6]. Th2 cells are important for different stages of foetus development but Th2 bias is also characteristic for allergic inflammation. It has been suggested that allergy related Th2 immune skewing may possibly be more beneficial than harmful for reproductive events. Allergic women become pregnant more easily, their children are at better general condition–they are heavier, and they are more mature according to gestational age [7]. However, it is unclear if an allergic status during pregnancy confers future allergy risk to the offspring or should we fight allergies during pregnancy.

Cytokine production during pregnancy is different for allergic and non-allergic women, with magnified Th2 effects in atopic subjects but response to allergens could occur even in pre-pregnancy non-allergic subjects [8, 9]. There are some studies comparing the allergen-specific IgE and total IgE levels before, during pregnancy and postpartum [10, 11]. They seem to be changing in time in allergic subjects, but the pattern is not clear and depends on the type of allergen [12]. Another theory suggest that total IgE is increasing with each pregnancy and higher levels are associated with multiple pregnancies [13].

Typically, women stop allergy treatments during pregnancy [14, 15]. If this behaviour is related to decreased symptom severity, or a fear of adverse events and birth defects, it is difficult to assume, but may aggravate the effect associated with maternal allergy. Increased allergic inflammation and an exacerbation of allergic disease, both conferring risk to the child, could be the consequences of the therapy cessation.

## Materials and methods

The aim of this study was to evaluate sIgE sensitisation to common allergens during pregnancy, and assess its relationship to symptoms and treatments of allergic conditions such as asthma, allergic rhinitis, atopic dermatitis and food allergy.

### Study participants

ELMA study is a prospective birth cohort starting from 2016 by selective recruitment of pregnant women, in the 3rd trimester, through the childbirth schools at obstetrics clinics and hospitals in Wroclaw, Poland. By August 2020 we enrolled 200 women. Their children are being followed up. Enrolment criteria were based on urban inhabitancy and the absence of reported pre-pregnancy obesity and metabolic complications such as diabetes and hypertension. Women who were currently smoking or exposed to second-hand smoking at home were excluded.

The current analysis is a part of project in which we investigate how early exposures, including maternal atopy and diet, impact the allergic disease susceptibility in offspring. The subpopulation of this group (n = 96) served as a case–control cohort for the epigenetic analysis of cord blood, which is a part of this study. That analysis and enrolment criteria were presented previously [16]. Here we focused on the sensitisation pattern and allergy outcome in ELMA’s pregnant women population.

### Assays

Peripheral blood samples were taken in order to evaluate serum levels of sIgE and total IgE. We tested all women for sIgE sensitivity to food and inhalant allergens. Specific IgE levels were estimated using the multi-parameter, quantitative panel POLYCHECK (Biocheck GmBh, Germany) with five calibrators. Assay data showed good concordance with UniCAP systems [17]. The test detected sIgE for a given allergen, over the range 0.1–100 kU/L. Participants were assigned as atopic when they were sensitised to at least one allergen, at a cut-off point of 0.35 kU/L. Additionally, we performed total IgE measurements using the DEIGE02 ELISA kit (Demeditec Diagnostics GmbH, Germany). Undetectable levels of total IgE was assigned as 0.0 kU/L.

### Questionnaires

Participants filled in questionnaires and were asked about allergies, DD (doctor diagnosed) allergic diseases, environmental factors such as pets at home, previous smoking habits, specific diets, supplements and anxiety. We queried allergies by asking; “Are you allergic?” Questions about allergic diseases were constructed as “Have you ever been diagnosed by a doctor for asthma, allergic rhinitis, atopic dermatitis, food allergies?” These questions are based on ECRHS (European Community Respiratory Health Survey) survey, modified with addition of doctor diagnosed term [18]. Also we asked about the worsening of allergic disease during pregnancy and medication used: “Have symptoms of asthma, allergic rhinitis, atopic dermatitis or food allergy get worse during pregnancy?” and “Have you been taking any medications for asthma or allergy?” (Additional file 1).

### Ethical considerations

All participants signed an informed consent prior to study commencement. Both blood sampling and questionnaire were done during one visit. The study was approved by the ethical committee of Wroclaw Medical University.

### Statistical analyses

Statistical analysis was performed with Statistica 13.1. For comparisons of categorical data, the chi^2^ test was implemented. For continuous variables, the U Mann–Whitney test was performed. We have assessed different phenotypes related to allergy: 1. Atopy–positive sIgE, 2. Reported allergy—based on the question “Are you allergic?” 3. Doctor diagnosed (DD) allergic disease—asthma, allergic rhinitis, atopic dermatitis or food allergy.

Regression analysis was used for total serum IgE as dependable variable. It was log transformed to meet normality assumptions. Independent variables in the model were age and prior pregnancies, as dichotomous variable and atopy, DD of allergic disease ever and gestational age–week of pregnancy et enrolment.

## Results

We assessed 200 pregnant women, with a mean age of 29 years (20–38), living in a metropolitan environment, with bad air quality (www.air-quality.com) [19]. 48.0% of them were sensitised to at least one allergen, at a cut-off point of 0.35 kU/L, and they were assigned as atopic. Atopic and non-atopic groups did not differ by age, smoking history and reporting allergy in child’s father. Atopic women less often had pets at home, and also had higher total IgE levels. Of 200 women, 190 (95%) were successfully followed to the 3rd month of child’s life when first medical examination was performed. At that visit, details regarding delivery were collected, such as birth weight, mode of od delivery and Apgar score. Children’s birth basic outcomes didn’t differ significantly between atopic and none-atopic women. Groups characteristics are shown in Table 1

**Table 1:** Patient characteristics in the atopic (sIgE positive) and non-atopic group (sIgE-negative)

	Atopic (sensitised) n = 96	Non-atopic n = 104	p value
Maternal age at enrolment (mean value in years ± SD)	29.62 (± 2.93)	30.27 (± 2.67)	0.15
Gestational age at enrolment (mean value in weeks ± SD)	34.46 (± 3.59)	34.27 (± 3.49)	0.63
History of smoking	36 (37.50%)	43 (41.75%)	0.50
Pets at home	24 (25.00%)	42 (40.38%)	**0.02**
Parity-first child	84 (87.50%)	93 (89.42%)	0.57
Tertiary education	88 (91.67%)	100 (96.15%)	0.32
Child’s father allergic	28 (29.17%)	35 (33.65%)	0.49
Decreased activity	57 (59.38%)	64 (62.1%)	0.57
Pregnancy perceived as stressful	31 (32.29%)	33 (31.73%)	0.33
Total IgE (mean IU/ml ± SD)	60.53 (± 78.39)	23.03 (± 32,16)	0.0000001

Children characteristics	Atopic mothers n = 92	Non-atopic mothers n = 98	
Gestational age at birth (mean value in weeks ± SD)	39.59 (± 1.28)	39.46 (± 1.33)	0.45
Apgar score	9.78 (± 0.84)	9.79 (± 0.72)	0.45
Birth weight (mean value in g ± SD)	3469.63 (± 501.81)	3433.93 (± 466.76)	0.46
Caesarean section	41 (44, 56%)	54 (51.92%)	0.15
Natural birth	51 (55.43%)	44 (44.89%)	

For comparisons of categorical data, the chi^2^ test was implemented. For continuous variables the U Mann–Whitney test was performed. Data are provided as numbers and frequencies–n (%) and mean ± standard deviation (SD)

In regression analysis regarding total IgE, we found out that atopy and self-reported DD allergic disease were significantly associated with higher levels of total IgE. In the non-atopic group parity was associated with increased total IgE (Table 2).

**Table 2:** Regression analysis for log10 transformed IgE as dependent variable and age, parity, BMI, height, week of pregnancy, atopy and self-reported doctor diagnosed (DD) allergic disease as independent variables

	Total IgE n = 190 R^2^ = 0.20 estimate	p value	Total IgE none-atopic n = 98 R^2^ = 0.06 estimate	p value
Age	0.058	0.41	0.07	0.54
Parity	0.04	0.57	0.24	0.026
Gestational age at enrolment	− 0.064	0.34	− 0.07	0.48
Pre-pregnancy BMI	0.05	0.45	0.05	0.63
Height	− 0.042	0.53	− 0.10	0.35
Atopy	0.37	**0.0000001**	n/a	n/a
Reported DD allergic disease	0.16	**0.02**	n/a	n/a

44.0% of women reported being allergic while asking—“are you allergic?”40.5% reported any DD allergic disease, 7.5% reported asthma, 21.5% reported allergic rhinitis, 11.5% reported atopic dermatitis and 18.5% reported food allergy. Allergic conditions worsened during pregnancy for a minority of cases–13.3% of all cases with asthma, 27.9% with allergic rhinitis and 30.4% with atopic dermatitis. A small proportion of women were taking medication for asthma or allergy (8.5%) Half of those not taking any medications reported worsening allergic disease. The differences were statistically significant (Table 3). In both atopic and none-atopic group, 1/3 of women perceived pregnancy as stressful situation. Majority (60.0%) limited their physical activity during pregnancy. Atopy was not significantly associated with neither factor.

**Table 3:** Self-reported allergic diseases in pregnant women n = 200, outlining the worsening cases and medication use

Outcome	n (%)	p
Any allergic disease	81 (40.50)	
DD asthma	15 (7.50)	
Worsening asthma	2 (1.00)	0.20
DD allergic rhinitis	43 (21.50)	
Worsening allergic rhinitis	12 (6.00)	0.24
DD atopic dermatitis	23 (11.50)	0.36
Worsening atopic dermatitis	7 (3.50)	
DD food allergy	37 (18.50)	0.09
Worsening food allergy	3 (1.50)	
Worsening any allergic disease	22 (27.16)	0.00011
Medication for allergy/asthma	17 (8.50)	

Fisher exact test was used for comparison of groups regarding medication and worsening of allergic disease

**Fig. 1:**
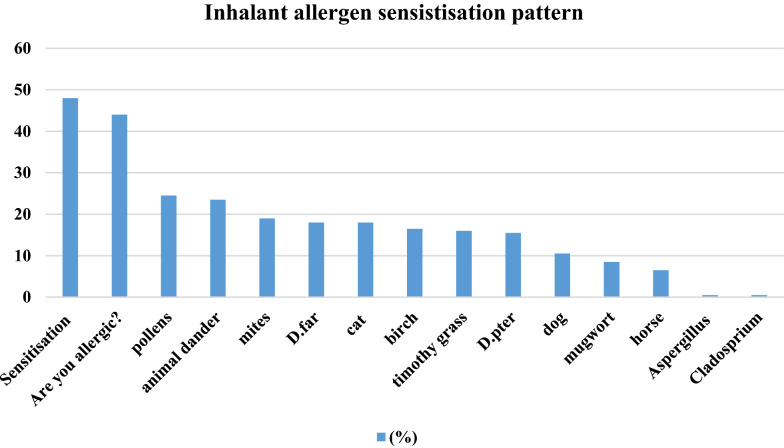
Inhalant sensitisation patterns in pregnant women, n = 200. Sensitisation was defined as at least one positive result with cut-off > 0,35kU/L for allergen-specific IgE

**Fig. 2:**
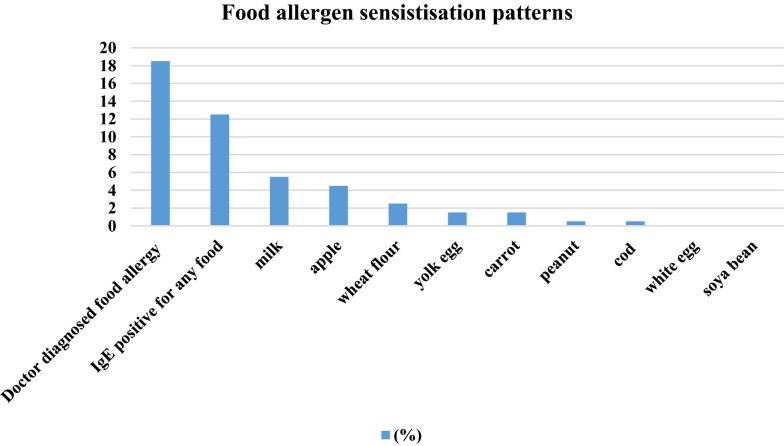
Food sensitisation patterns in pregnant women, n = 200

The most common sensitising inhalant allergens were pollen (24.5%) and animal dander (23.5%). Approximately 12.5% of women were sensitised to food; the most common were cow’s milk (5.5%) and apple (4.5%) (Figs. 1, 2).

**Fig. 3:**
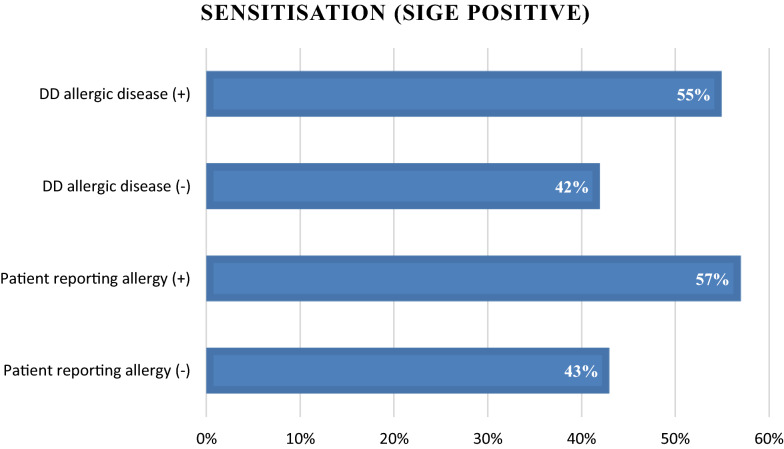
Allergen sensitisation in relation to DD allergic disease–asthma, allergic rhinitis, atopic dermatitis or food allergy and self-reported allergies, n = 96 for those sensitised to any inhalant or food allergen. Sensitisation was defined as at least one positive result with cut-off > 0.35kU/L for allergen-specific IgE

While comparing groups reporting allergic disease and sensitised (atopic) 42–43% in atopic group were not reporting an allergy, either doctor diagnosed or self-perceived (Fig. 3). According to self-reported questionnaires, but also sensitisation pattern, food allergies had a relatively high prevalence in our group, 18.5% and 12.5% respectively. Only 24.3% of women reporting doctor diagnosed food allergy presented a positive sIgE response for any food.

## Discussion

We presented here a spectrum of allergic sensitisation and allergy diagnoses in the group of pregnant women living in certain environment. These results are part of an ELMA study designed to evaluate maternal atopy and diet on the methylation pattern in neonatal cord blood, and latter development of allergy in offspring. Since the study is ongoing, this data relates only to maternal factors. According to the protocol, all women enrolled had a normal pregnancy with no diagnosis of pre-pregnancy obesity, gestational diabetes or chronic hypertension, they did not smoke and were not exposed to ETS and lived in an urban area.

IgE sensitisation to common allergens was quite common in our study group. Approximately half of women were sensitised to at least one allergen, and they were assigned as atopic. This proportion is similar to the European atopy prevalence in young adults (< 45 years old), which is 51% [20]. In those women assigned as atopic 42% were not diagnosed before pregnancy–they answered “no” for question “Have you ever been diagnosed by doctor for…’. This proportion is similar while comparing self-reported allergy (Are you allergic?)–43% answered “no”. An explanation could be that there was an asymptomatic sensitisation present in these women. However because we didn’t ask directly for allergic symptoms, such as wheezing or runny nose, this conclusion seems to be uncertain. In our previous study we have shown that the methylation pattern in cord blood is related to maternal atopy–defined as sensitisation to at least one allergen [16].

Few studies have focussed on allergic sensitisation during pregnancy [21, 22]. In one study, house dust mites appeared to sensitise pregnant women predominantly with high prevalence [23]. In our group, the most common sensitising airborne allergens were pollens (24.5%), but numbers for pollen, dust mites (19.0%) and animal (23.5%) sensitisation were quiet similar. In some studies, differences were observed among asthmatic patients living in urban and rural areas in regard to mite sensitisation, with the latter showing less positive results. This suggests a high impact of increased exposure to indoor allergens in an urbanised area, related mainly to life-style factors [24, 25]. However, the process of air pollution in big cities also causes the modification of plant-derived pollens so that they have increased ability to induce sensitisation, resulting in a higher incidence of pollen-induced respiratory allergy in urban areas in comparison to rural ones [1].

There is some evidence that even while not presenting with evident allergic symptoms during pregnancy period, asymptomatic sensitisation could pose risks to the unborn child. IgE transfers easily from mother to child via placenta [26]. As a consequences it triggers allergic cascade with Th2 lymphocytes, crucial cells for allergic inflammation. Th2 cells also transfer via placenta and had been relating as conferring maternal transfer of asthma risk. In murine model of maternal asthma, IL-4 blockade—key Th2 cytokine, decreases the conferring risk in offspring, whereas IFN gamma, known for its anti-allergic function, has the opposite effect [5]. What’s more both allergens and allergen-specific IgG can be delivered trans-placental to the murine and human foetus [27].

40.5% of women in our group reported DD allergic conditions. The prevalence rates for asthma, allergic rhinitis and atopic dermatitis observed in our study, i.e. 7.5%, 21.5%, 11.5% were similar to data in European adults; 8.5%, 23–30%, 2.2–17.6%, respectively [1, 28–30]. There are also consistent data available for the Polish population for both urban and rural areas (urban: asthma 4.0%, allergic rhinitis 22.1%, eczema 34.9%; rural: asthma 1.9%, allergic rhinitis 12.8%, eczema 24.1%) [31, 32]. We believe therefore that results from our study are good estimate for general population.

Allergy got worse during pregnancy in 27% of participants, and only one fifth of allergic women were taking allergy medications, which impacted the disease control. In other studies on asthma and allergic rhinitis in pregnancy the proportions were similar; approximately 33% of pregnant asthma patients suffered with symptom deterioration, one third were stable and the remainder were better [33]. For allergic rhinitis, the disease could go either way [29]. Atopic dermatitis is worsening in 20% during pregnancy according to other studies [34]. Women with asthma tend to decrease their medication–in 23% for inhaled corticosteroid, in 13% for short acting beta agonists in 54% for rescue corticosteroid. 1/3 discontinue asthma medication without consulting a doctor [35]. On the other hand, during 9 months of pregnancy, women are taking on average 11 different medication, and antihistaminic are the most commonly used. So far for II generation antihistamines, which are recommended for use in pregnancy, are no association with birth defects [36].

In general, pregnant women tend to avoid any medication, even though four out of five is prescribed with some [14]. We assume avoiding anti-allergic medicines reflects anxiety regarding adverse events during pregnancy, as opposed to the mildness of the disease (where there is no need to apply any treatment). Women prefer to suffer a little bit with itchy eyes and runny nose than risk, in their opinion, child’s health. One from few studies of asthma during pregnancy revealed that the fear for ICS side effect for foetus was one of the main factors interfering with asthma control [37]. There issparse evidence from studies of atopic dermatitis and allergic rhinitis during pregnancy [34].

**Fig. 4:**
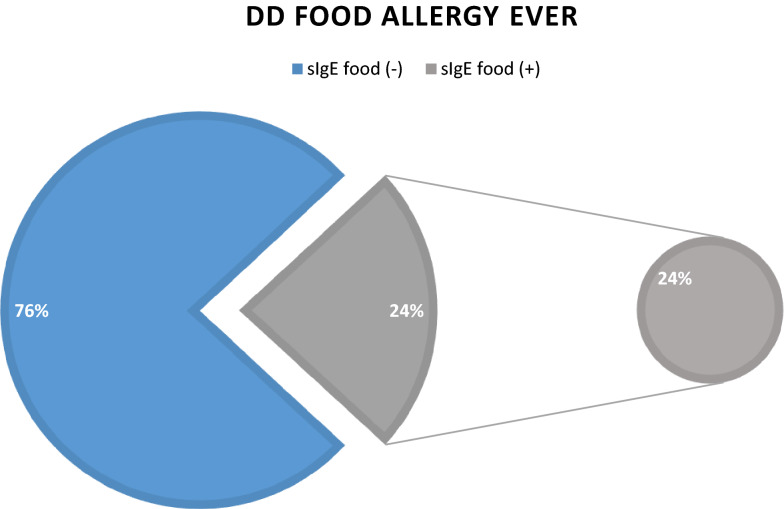
Doctor diagnosis food allergy in relation to food sensitisation (sIgE positive), n = 37 reported DD food allergy

We observed that self-reported food allergies were common in our group–18.5% of the total. However, in only 24.3% of cases these reported DD food allergy ever, we observed an IgE mediated input (Fig. 4). Also among women not reporting any food allergies, 9.8% were positive for food specific IgE. In these subjects “food IgE positive” and “self-reported negative”, we presume sensitisation is either not diagnosed or asymptomatic.

The prevalence of sIgE sensitisation for different food allergens in our group—12.5%, with 5.5% sensitised to cow’s milk, was high in comparison to other studies. In Europe, the prevalence of sIgE sensitisation to any food is 4.1%, for self-reported food allergies, it is 17% [38]. In US in adults self-reported food allergy is as common as 10%, with milk allergy 1.9% [39]. In our study this high prevalence of food sensitisation could be explained partially by cross-reactivity (15% sensitised to birch were sensitised to apple), but milk sensitisation is unexpected [40]. Our results raises the questions; if pregnancy facilitate sensitisation to food, or more specifically milk and whether there are any consequences of this susceptibility to offspring. There are very limited resources in literature describing adult-onset food allergies [41, 42], and to our knowledge none describing this phenomenon in pregnancy.

There are reports which have suggested thatacid-suppressing drugs such PPI (proton pump inhibitors), and histamine 2 (H2) receptor antagonistsfacilitate de novo allergic sensitisation in adults [43]. In our group, women were asked about any medication used in pregnancy, and they did not report any anti-acid treatments, so we can not explain the increased prevalence of food allergy in our study by this factor.

We assume that the clinical implication of this observation could be a possible risk for allergy outcomes in offspring. Since it was not the aim of this analysis, and the data from the literature are scarce, it requires further investigation. Some evidence of such an association is provided by animal studies [44, 45].

Total IgE has being estimated as a marker of atopy. We observed increased levels of total IgE in atopic vs none-atopic subjects in our study, which is consistent with other reports. There were no differences with respect to maternal age, but in none-atopic group parity was associated with increased values, similarly to other studies [13]. It was shown previously that levels of total but not specific IgE are increasing during early pregnancy in sensitised women with allergic symptoms, in comparison to period 12 months after the delivery. This suggest that allergy is associated with increased Th2 bias during pregnancy [10]. It was also revealed that the pattern for sIgE, in the course of pregnancy could depend on the type of allergen, with constant increase over time only for some of them [12].

Atopic women were not perceiving their pregnancies as stressful more often than none-atopic women. So atopy couldn’t be regarded as anxiety trigger. It seems plausible that every condition regarding maternal health is some kind of stress for future mother, but in our study 1/3 of women considered pregnancy as stressful regardless to atopy status.

Additionally about 60% of women in both group reported decrease physical activity during pregnancy. The number is similar to other reports. In US only 23–29% women at any gestational stage met the minimum physical activity guidelines [46]. This important lifestyle factor affects both mother wellbeing, with reduced risk of post-partum depression in active women [47] and also child’s health with decreased risk of unfavourable pregnancy outcomes, such as preterm birth, prolonged delivery and caesarean section [48]. Atopy didn’t have significant impact on physical activity in our study, but 80% women with diagnosed asthma limited their physical activity.

We didn’t observed any differences for birth outcomes such as weight, Apgar score and Caesarean section frequency and gestational age between atopic and none-atopic women. These observations are similar to other findings, where maternal atopy didn’t confer any risk for unfavourable birth outcomes [49].

Our study has some limitations. First, we lack a control group of non-pregnant women living in urban areas. We can only compare our results to those of epidemiological studies in a similar age group. For those, there are results available for both urban and rural areas. Also, we did not check for sensitisation before pregnancy and post-partum, so it is difficult to estimate whether this process occurred in pregnancy or if it is transient. The strength of our study is carefully designed homogenous study population which defined risk factors. We asked in questionnaires about the allergic diseases and also performed atopy screening by sIgE. There are not many studies regarding allergy in pregnancy, some of them are based on the population born some time ago. With increasing prevalence of allergies in young people and changing environment, new data are needed.

## Conclusions

Allergic sensitization is a common phenomenon in pregnancy. Only partially this is related to pre-existing diagnosis of allergic disease. Some sensitizations could be asymptomatic. Further studies should investigate if sensitization in mothers confers a risk of immune alterations in their unborn children.

## Supplementary Information

**Additional file 1.** Study questionnaire

## Data Availability

Data of participating subjects are confidential. The datasets used and/or analysed during the current study are available from the corresponding author on reasonable request.

## References

[1] Ring J, Akdis CA, Agache I. What is allergy, Global Atlas of Allergy. Eur Acad Allergy Clin Immunol. 2014; 2–3. http://www.eaaci.org

[2] Trønnes H, Wilcox AJ, Markestad T, Tollånes MC, Lie RT, Moster D (2014). Associations of maternal atopic diseases with adverse pregnancy outcomes: a national cohort study. Paediatr Perinat Epidemiol..

[3] Strachan DP, Wickens K, Crane J, Pearce N, Beasley R (2000). Family size, infection and atopy: the first decade of the “hygiene hypothesis” The magnitude of the effect of smaller family sizes on the increase in the prevalence of asthma and hay fever in the United Kingdom and New Zealand. Thorax.

[4] Hvas Mortensen L, Helweg-Larsen K, Nybo Andersen A-M (2011). Socioeconomic differences in perinatal health and disease. Scand J Public Health..

[5] Hubeau C, Apostolou I, Kobzik L (2006). Adoptively transferred allergen-specific T cells cause maternal transmission of asthma risk. Am J Pathol..

[6] Lauzon-Joset JF, Mincham KT, Abad AP, Short BP, Holt PG, Strickland DH (2019). Oestrogen amplifies pre-existing atopy-associated Th2 bias in an experimental asthma model. Clin Exp Allergy..

[7] Savilahti E, Siltanen M, Pekkanen J, Kajosaari M (2004). Mothers of very low birth weight infants have less atopy than mothers of full-term infants. Clin Exp Allergy..

[8] Abelius MS, Jedenfalk M, Ernerudh J, Janefjord C, Berg G, Matthiesen L, et al. Pregnancy modulates the allergen-induced cytokine production differently in allergic and non-allergic women. Pediatr Allergy Immunol. 2017; 28: 818–24. http://doi.wiley.com/10.1111/pai.12802 10.1111/pai.12802 28892576

[9] Breckler LA, Hale J, Jung W, Westcott L, Dunstan JA, Thornton CA (2010). Modulation of in vivo and in vitro cytokine production over the course of pregnancy in allergic and non-allergic mothers. Pediatr Allergy Immunol Pediatr Allergy Immunol.

[10] Sandberg M, Frykman A, Jonsson Y, Persson M, Ernerudh J, Berg G (2009). Total and allergen-specific IgE levels during and after pregnancy in relation to maternal allergy. J Reprod Immunol.

[11] Bahna SL, Woo CK, Manuel PV, Guarderas JC (2011). Serum total IgE level during pregnancy and postpartum. Allergol Immunopathol. SEICAP.

[12] Hedman AM, Lundholm C, Scheynius A, Alm J, Andolf E, Pershagen G, et al. Allergen-specific IgE over time in women before, during and after pregnancy. Allergy Eur J Allergy Clin Immunol. 2019; 74: 625–8. https://onlinelibrary.wiley.com/doi/abs/10.1111/all.13662 10.1111/all.13662 30408222

[13] Rivara AC, Miller EM (2017). Pregnancy and immune stimulation: re-imagining the fetus as parasite to understand age-related immune system changes in US women. Am J Hum Biol..

[14] Stock SJE, Norman JE. Medicines in pregnancy. F1000Research. F1000 Research Ltd; 2019; 8: 911. https://f1000research.com/articles/8-911/v1. Accessed 11 Apr 2020

[15] Namazy JA, Schatz M (2016). The treatment of allergic respiratory disease during pregnancy. J Investig Allergol Clin Immunol.

[16] Danielewicz H, Gurgul A, Dębińska A, Myszczyszyn G, Szmatoła T, Myszkal A (2020). Maternal atopy and offspring epigenome-wide methylation signature. Epigenetics.

[17] Jeong S, Jang G-C, Cho NJ, Han MS, Kim HS, Sun JY (2012). Analysis of polycheck allergy results of the recent two years: comparison with skin prick test and immunoCAP. Lab Med Online.

[18] Janson C, Anto J, Burney P, Chinn S, De Marco R, Heinrich J (2001). The European community respiratory health survey: what are the main results so far?. Eur Respirat J..

[19] Statystyczny PGU. Rocznik statystyczny województw. Papier . 2019; http://scholar.google.com/scholar?hl=en&btnG=Search&q=intitle:Rocznik+statystyczny+województw#0

[20] Newson RB, Van Ree R, Forsberg B, Janson C, Lötvall J, Dahlén SE (2014). Geographical variation in the prevalence of sensitization to common aeroallergens in adults: the GA2LEN survey. Allergy Eur J Allergy Clin Immunol.

[21] Ryabova MA, Lavrova OV, Shumilova NA, Pestakova LV (2018). Allergic rhinitis in the pregnant women. Vestn Otorinolaringol..

[22] Perry LM, Ownby DR, Wegienka GR, Peterson EL, Woodcroft KJ, Joseph CL (2009). Differences in total and allergen specific IgE during pregnancy compared with 1 month and 1 year post partum. Ann Allergy Asthma Immunol..

[23] Yeh CC, Wu KG, Wang PH (2018). High prevalence of IgE sensitization against house dust mites in pregnant women. Medicine.

[24] Bibi H, Shoseyov D, Feigenbaum D, Nir P, Shiachi R, Scharff S (2002). Comparison of positive allergy skin tests among asthmatic children from rural and urban areas living within small geographic area. Ann Allergy Asthma Immunol..

[25] Pawankar R, Canonica GW, Holgate ST, Lockey RF, editors. The WAO white book on allergy. World Allergy Organization. 2011. pp. 1–216. http://www.worldallergy.org/UserFiles/file/WAO-White-Book-on-Allergy.pdf.

[26] Bundhoo A, Paveglio S, Rafti E, Dhongade A, Blumberg RS, Matson AP (2015). Evidence that FcRn mediates the transplacental passage of maternal IgE in the form of IgG anti-IgE/IgE immune complexes. Clin Exp Allergy.

[27] Macchiaverni P, Arslanian C, Frazão JB, Palmeira P, Russo M, Verhasselt V (2011). Mother to child transfer of IgG and iga antibodies against dermatophagoides pteronyssinus. Scand J Immunol..

[28] Jarvis D, Newson R, Lotvall J, Hastan D, Tomassen P, Keil T (2012). Asthma in adults and its association with chronic rhinosinusitis: the GA2LEN survey in Europe. Allergy.

[29] Dhillon RS, Fairley JW (1989). Clinical features of allergic rhinitis. Mult Quest Otolaryngol..

[30] Harrop J, Chinn S, Verlato G, Olivieri M, Norbäck D, Wjst M (2007). Eczema, atopy and allergen exposure in adults: a population-based study. Clin Exp Allergy.

[31] Samoliński B, Sybilski AJ, Raciborski F, Tomaszewska A, Samel-Kowalik P, Walkiewicz A (2009). Prevalence of rhinitis in polish population according to the ECAP (epidemiology of allergic disorders in Poland) study. Otolaryngol Pol..

[32] Krzych-Fałta E, Furmańczyk K, Piekarska B, Tomaszewska A, Sybilski A, Samoliński BK (2016). Allergies in urban versus countryside settings in poland as part of the epidemiology of the allergic diseases in poland (ECAP) study-challenge the early differential diagnosis. Postep Dermatologii i Alergol..

[33] Global initiative for asthma (2019). Asthma management and prevention, 2019. Pract Nurse..

[34] Vestergaard C, Wollenberg A, Barbarot S, Christen-Zaech S, Deleuran M, Spuls P (2019). European task force on atopic dermatitis position paper: treatment of parental atopic dermatitis during preconception, pregnancy and lactation period. J Eur Acad Dermatol Venereol.

[35] Pali-Schöll I, Namazy J, Jensen-Jarolim E (2017). Allergic diseases and asthma in pregnancy, a secondary publication. World Allergy Organ J..

[36] Dávila I, del Cuvillo A, Mullol J, Jáuregui I, Bartra J, Ferrer M (2013). Use of second generation H1 antihistamines in special situations. J Investig Allergol Clin Immunol.

[37] Ibrahim WH, Rasul F, Ahmad M, Bajwa AS, Alamlih LI, El Arabi AM, et al. Asthma knowledge, care, and outcome during pregnancy: The QAKCOP study. Chron Respir Dis. SAGE Publications Ltd; 16:147997231876771. 10.1177/1479972318767719 PMC6302965 29621888

[38] Muraro A, Werfel T, Hoffmann-Sommergruber K, Roberts G, Beyer K, Bindslev-Jensen C (2014). EAACI food allergy and anaphylaxis guidelines: diagnosis and management of food allergy. Allergy Eur J Allergy Clin Immunol.

[39] Gupta RS, Warren CM, Smith BM, Jiang J, Blumenstock JA, Davis MM (2019). Prevalence And Severity Of Food Allergies Among US adults. JAMA Netw Open.

[40] Lyons SA, Burney PGJ, Ballmer-Weber BK, Fernandez-Rivas M, Barreales L, Clausen M (2019). Food allergy in adults: substantial variation in prevalence and causative foods across Europe. J Allergy Clin Immunol Pract..

[41] Lopes JP, Sicherer S (2020). Food allergy: epidemiology, pathogenesis, diagnosis, prevention, and treatment. Curr Opin Immunol..

[42] Sicherer SH, Sampson HA (2018). Food allergy: a review and update on epidemiology, pathogenesis, diagnosis, prevention, and management. J Allergy Clin Immunol..

[43] Schöll I, Ackermann U, Özdemir C, Blümer N, Dicke T, Sel S (2007). Anti-ulcer treatment during pregnancy induces food allergy in mouse mothers and a Th2-bias in their offspring. FASEB J..

[44] Kopp MV, Zehle C, Pichler J, Szépfalusi Z, Moseler M, Deichmann K (2001). Allergen-specific T cell reactivity in cord blood: the influence of maternal cytokine production. Clin Exp Allergy.

[45] Song Y, Liu C, Hui Y, Srivastava K, Zhou Z, Chen J (2014). Maternal allergy increases susceptibility to offspring allergy in association with TH2-biased epigenetic alterations in a mouse model of peanut allergy. J Allergy Clin Immunol..

[46] Dipietro L, Evenson KR, Bloodgood B, Sprow K, Troiano RP, Piercy KL (2019). Benefits of physical activity during pregnancy and postpartum: an umbrella review. Med Sci Sports Exerc.

[47] Nakamura A, van der Waerden J, Melchior M, Bolze C, El-Khoury F, Pryor L (2019). Physical activity during pregnancy and postpartum depression: Systematic review and meta-analysis. J Affect Disord..

[48] de Oliveira CS, dos Imakawa TS, Moisés ECD (2017). Physical activity during pregnancy: recommendations and assessment tools. Rev Bras Ginecol e Obstet..

[49] Johnson A, Mason T, Kirby RS, Ledford D, Salihu HM (2017). Examining the association between maternal atopy and birth outcomes using a retrospective cohort in the southeastern region of the USA. BMJ Open.

