# Supplementary material for: Sensitisation patterns and allergy outcomes in pregnant women living in the urban area
Source: Allergy Asthma Clin Immunol. 2021 May 10;17:46. doi: 10.1186/s13223-021-00547-0 (PMC8111908; doi:10.1186/s13223-021-00547-0)
Supplement: Supplementary file 1 — Additional file 1. Study questionnaire [file 13223_2021_547_MOESM1_ESM.docx]

**
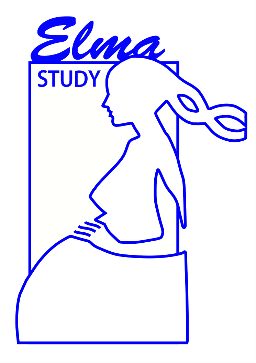
Respondent code:**

**Date of survey:**

**Atopic questionnaire**

**Please kindly answer the questionnaire questions about your health.**

**The correct answer should be marked with an "X"**

**The date of the last menstruation:**

**Week/month of pregnancy:**

**Due date:**

**Age:**

|  | YES | NO |
| --- | --- | --- |
| 1. Are you allergic? |  |  |
| 1a. If yes, please mention allergens you are sensitised to: | | |

| 2. Have you ever been diagnosed by a doctor with asthma? |  |  |
| --- | --- | --- |
| 2a.  If YES, have asthma symptoms get worse during pregnancy? |  |  |
| 3. Have you ever been diagnosed by a doctor with allergic rhinitis? |  |  |
| 3a. If YES, have the symptoms of allergic rhinitis get worse during pregnancy? |  |  |
| 4. Have you ever been diagnosed by a doctor with atopic dermatitis? |  |  |
| 4a. If YES, have the symptoms of atopic dermatitis get worse during pregnancy? |  |  |
| 5. Have you ever been diagnosed by a doctor with food allergy? |  |  |
| 5a. If YES, have the symptoms of food allergy get worse during pregnancy? |  |  |
| 6. Have you taken or are you taking any anti-allergic or anti-asthmatic medicines during pregnancy? |  |  |
| 6a. If YES, please list the names and dosages: | | |
| 7. Is the child's father allergic? |  |  |
| 7a. Does the child's father have asthma? |  |  |
| 7b. Did the child's father smoke cigarettes? |  |  |
| 8. Are there pets in the house? |  |  |
| 8a. If YES, please mention: | | |
| 9. Have you ever smoked cigarettes? |  |  |
| 9a. If YES please specify the period: | | |
| 10. Have your parents ever smoked cigarettes? |  |  |
|  | YES | NO |
| 11. Are you currently on a specialized diet – hypoallergenic, low-fat or other from medical indication? |  |  |
| 11a. If YES what is the diet? | | |
| 12. Do you take dietary supplements during pregnancy? |  |  |
| 12a. If YES, please list the names: | | |
| 13. Do you take vitamin preparations during pregnancy? |  |  |
| 13a. If YES, please list the names and dosages: | | |
| 14. Are you on sick leave during pregnancy? |  |  |
| 14a. If you work professionally during pregnancy, do you find your work stressful? |  |  |
| 15. Is pregnancy a stressful situation for you? |  |  |
| 16. Have you restricted physical activity during pregnancy? |  |  |
| 17. Do you currently attend or did you attend fitness classes for pregnant women? |  |  |
| 18. Do you have children (apart from your current pregnancy)? |  |  |
| 18a. If YES please indicate their gender and age: | | |
| 19. What is your education: primary, higher, secondary? – please enter: | | |

Thank you for your time and attention ☺
